# Sex Differences in Colorectal Cancer Survival: Population-Based Analysis of 164,996 Colorectal Cancer Patients in Germany

**DOI:** 10.1371/journal.pone.0068077

**Published:** 2013-07-05

**Authors:** Ondrej Majek, Adam Gondos, Lina Jansen, Katharina Emrich, Bernd Holleczek, Alexander Katalinic, Alice Nennecke, Andrea Eberle, Hermann Brenner, the GEKID Cancer Survival Working Group

**Affiliations:** 1 Division of Clinical Epidemiology and Aging Research, German Cancer Research Center, Heidelberg, Germany; 2 Institute of Biostatistics and Analyses, Masaryk University, Brno, Czech Republic; 3 Cancer Registry of Rhineland-Palatinate, Institute for Medical Biostatistics, Epidemiology and Informatics, University Medical Center, Johannes Gutenberg University Mainz, Mainz, Germany; 4 Saarland Cancer Registry, Saarbrücken, Germany; 5 Cancer Registry of Schleswig-Holstein, Institute of Cancer Epidemiology, University of Lübeck, Lübeck, Germany; 6 Hamburg Cancer Registry, Ministry for Health and Consumer Protection, Hamburg, Germany; 7 Cancer Registry of Bremen, Leibniz-Institute for Prevention Research and Epidemiology - BIPS, Bremen, Germany; Sapporo Medical University, Japan

## Abstract

Risk of colorectal cancer (CRC) is considerably higher in men compared to women; however, there is inconclusive evidence of sex differences in CRC prognosis. We aimed to assess and explain sex differences in 5-year relative survival using standard and model-based period analysis among 164,996 patients diagnosed with CRC from 1997 to 2006 and reported to 11 German cancer registries covering a population of 33 million inhabitants. Age-adjusted 5-year relative survival was higher in women (64.5% vs. 61.9%, *P*<0.0001). A substantial survival advantage of women was confirmed in multivariate analysis after adjusting for CRC stage and subsite in subjects under 65 years of age (relative excess risk, RER 0.86, 95% CI 0.82–0.90), but not in older subjects (RER 1.01, 95% CI 0.98–1.04); this pattern was similar in the 1st and in the 2nd to 5th year after diagnosis. The survival advantage of women varied by CRC stage and age and was most pronounced for localized disease (RERs 0.59–0.88 in various age subgroups) and in patients under 45 years of age (RERs 0.59, 0.72 and 0.76 in patients with localized, regional or advanced disease, respectively). On the contrary, sex differences in survival did not vary by location of CRC. In conclusion, our large population-based study confirmed a survival advantage of female compared to male CRC patients, most notably in young and middle aged patients and patients with localized disease. The effect of sex hormones, either endogenous or through hormonal replacement therapy, might be the most plausible explanation for the observed patterns.

## Introduction

Colorectal cancer (CRC) is the third most common cancer and the fourth most common cancer cause of death, accounting for more than 600,000 deaths per year globally [1]. In most countries, incidence and mortality rates are considerably higher in men than in women [2]. On the other hand, findings regarding sex differences in prognosis have been less consistent. Several studies reported superior survival in females [3], [4], [5]; however, other studies did not report any difference [6].

Two recent studies examined potential variation of sex differences in survival of CRC patients by age. Whereas younger women exhibited better survival than younger men, an opposite pattern was seen among older patients [7], [8]. As the age cut off (around 50 years) was chosen as a surrogate for natural menopause, it was hypothesized that the survival advantage of female patients at younger age could be partially explained by favourable effect of endogenous female sex hormones.

Other factors potentially accounting for sex differences in survival are differences in use of screening offers and stage at diagnosis, and differences in site distribution of CRC. Offer and use of screening examinations vary between countries [9]. In Germany, screening by faecal occult blood test (FOBT) has been offered since 1977. Since 2002, colonoscopy has been offered as primary screening examination from age 55 on. Participation rates have been higher in women than in men for both FOBT and screening colonoscopy, especially in younger age groups, which may have contributed to a higher proportion of early stages and better prognosis [10], [11]. As a different mediator of survival advantage in women, higher postoperative morbidity in men leading to early deaths unrelated to CRC was hypothesized [12]. On the other hand, CRC is more often located in the proximal colon among women [13]; this was reported to be a rather unfavourable prognostic factor [14], [15].

We aimed to compare survival from CRC between men and women and to explore potential reasons for sex differences in a large population-based cancer survival study from Germany.

## Materials and Methods

### Sources of Data

As this was a retrospective epidemiological study based on anonymised cancer registry data, neither approval of the ethics committee nor patient informed consent was needed.

This analysis is part of a collaborative project aiming for comprehensive monitoring of cancer survival in Germany. Full details were described elsewhere [16]. In brief, German cancer registries from 13 of 16 federal states submitted data for the study. Only areas with estimated completeness of cancer registration over 80% in the period 2004–2006 and reasonably low proportions of death certificate only (DCO) cases (under 20% throughout the study period or constantly decreasing to levels below 20% at the end of study period) were considered for the analysis. Subsequently, only data of selected districts from four states were included in the analyses. We eventually utilized data from 11 cancer registries covering a population of 33 million inhabitants.

The database for this analysis included patients with a primary invasive CRC (ICD-10 C18–C20) at the age of 15 years or older in 1997–2006. We excluded cases notified by DCO. For the stage-specific analysis, stage grouping according to ENCR recommendations (localized, regional, and advanced cancer) was used [17].

Tumour site and morphology of tumours were coded according to ICD-O-3 [18]. Right colon included caecum (topography code C18.0), ascending colon (C18.2), hepatic flexure (C18.3), and transverse colon (C18.4). Left colon included splenic flexure (C18.5), descending (C18.6) and sigmoid (C18.7) colon. Cancers of appendix, overlapping lesions and cancers within unspecified colonic subsite were included in the ‘Colon – unspecified/other’ group. For the purpose of our analysis, rectosigmoid junction (C19) and rectum (C20) were considered together.

### Statistical Methods

To quantify excess mortality due to cancer, relative survival is commonly computed in population based cancer survival studies. It is derived as the ratio of the observed survival of cancer patients and the expected survival of the underlying general population [19]. In our analysis, expected survival was estimated by the so-called Ederer II method [20] using life tables stratified by age, sex, calendar period and federal state as obtained from participating cancer registries and the German Federal Statistical Office. To allow for comparisons between subgroups or populations with potentially different age distribution, age adjustment was done using the International Cancer Survival Standards proposed by Corazziari et al. [21]. Period analysis [22] was employed to provide the most up-to-date estimates of 5-year relative survival. The period analysis included survival experience in the period from 2002 to 2006; all observations were left truncated at the beginning of 2002 and right censored at the end of 2006. Extensive empirical evaluations have shown that period analysis provides 5-year survival estimates that are very close to 5-year survival later observed for the patients diagnosed within the period of investigation [23], [24] and is now widely used in cancer registry based survival analyses (e.g. [25], [26], [27]). To evaluate the contribution of early postoperative mortality and later mortality to sex differences, 1-year relative mortality as well as relative survival at 5 years after diagnosis conditional on surviving 1 year was calculated in addition to overall 5-year relative survival. Standard errors were estimated using Greenwood’s formula, sex differences were tested for statistical significance by model-based period analysis [28].

Model-based period analysis was also used to perform multivariate analysis [29]. Briefly, numbers of deaths were modelled as a function of the categorical variables year of follow up, age (15–44, 45–54, 55–64, 65–74, 75+), sex, CRC stage (localized, regional, advanced, not reported) and subsite (left colon, right colon, colon – unspecified/other, rectum) with the logarithm of the person-years at risk as an offset. Estimates of relative excess risk (RER) of dying from CRC (women vs. men) and a *P*-value for the difference in excess risks between sexes were derived using described statistical model with different interaction terms included among covariates. To assess potential effect of age on sex differences, we first included an interaction term of sex and age. To assess whether women’s advantage differ between subgroups by stage or subsite, we fitted models moreover including interactions of sex and stage or sex and subsite. To estimate the log(RER) for a particular age-stage subgroup, we calculated the sum of following terms: the regression coefficient for the effect of female sex, the respective regression coefficient for the interaction of female sex and particular age group, and the respective regression coefficient for the interaction of female sex and particular stage. An analogous procedure was applied to estimate the log(RER) for a particular age-subsite subgroup.

All calculations were carried out by SAS software version 9.3 (SAS Institute, Cary, NC), using a publicly available macro for period analysis [30] and its adaptation for modelled period analysis [28] and multivariate analysis.

## Results

After exclusion of 27,076 DCO notifications (14.1%), records of 164,996 CRC patients from 11 German cancer registries were retained for analysis. The vast majority of cancers (97.8%) were microscopically verified.

Table 1 shows distributions of male and female patients according to age and CRC stage and subsite. Slightly more men (86,704) than women (78,292) were diagnosed with CRC in 1997–2006. Women were diagnosed at later age (median age 73 years vs. 68 years for males) and subsequently have a higher proportion of DCO cases than men (16.3% vs. 11.8%, data not shown) and also a higher proportion of cases with unknown stage (43.5% vs. 41.0%). Stage was reported for 95,422 cancer patients, of whom 44.2%, 27.7%, and 28.1% were diagnosed in localized, regional, and advanced stage. Proportions of localized, regional and advanced cancers among those with known stage were similar in men (44.5%, 27.0% and 28.5%) and women (44.0%, 28.5% and 27.6%). On the other hand, right colon tumours were more common in females (30.8% of CRCs in women vs. 22.5% in men) and rectal tumours were more common in males (40.3% of CRCs in men vs. 32.5% in women).

**Table 1:** Sex differences in distributions of age, stage and subsite in colorectal cancer patients diagnosed between 1997 and 2006.

	Men		Women	
	*N*	%*	*N*	%*
**Age**				
15–44	2,159	2.5	1,954	2.5
45–49	2,395	2.8	1,812	2.3
50–54	4,378	5.0	3,014	3.8
55–59	7,857	9.1	4,903	6.3
60–64	13,925	16.1	8,297	10.6
65–69	17,034	19.6	10,707	13.7
70–74	15,726	18.1	12,117	15.5
75–79	12,382	14.3	14,267	18.2
80–84	7,043	8.1	11,930	15.2
85+	3,805	4.4	9,291	11.9
**Stage**				
Localized	22,773	26.3	19,451	24.8
Regional	13,832	16.0	12,593	16.1
Advanced	14,573	16.8	12,200	15.6
Not reported	35,526	41.0	34,048	43.5
**Subsite**				
Right colon	19,520	22.5	24,094	30.8
Left colon	25,393	29.3	21,703	27.7
Colon-unspecified/other	6,821	7.9	7,084	9.0
Rectum	34,970	40.3	25,411	32.5
**Total**	**86,704**		**78,292**	

* Percentages represent proportion of all males/females.

As shown in Table 2, age-adjusted 5-year relative survival was higher in women (64.5% vs. 61.9%, *P*-value<0.0001). Age-specific 5-year relative survival decreased with increasing age in both men (65.5% in youngest to 54.8% in oldest) and women (71.8% in youngest to 56.7% in oldest). The survival advantage of women was largest in patients below 45 years of age (6.3 percent units) and between 50 and 64 years of age (between 3.8 and 6.0 percent units).

**Table 2:** Age-specific 5-year relative survival of colorectal cancer patients in period 2002–2006 by sex.

	Men		Women		Women - Men	
Age	RS, %	SE	RS, %	SE	Difference	*P-*value
15–44	65.5	1.5	71.8	1.5	6.3	<0.001
45–49	66.0	1.4	65.6	1.6	−0.4	0.95
50–54	62.8	1.1	68.2	1.3	5.4	<0.001
55–59	64.9	0.8	68.7	1.0	3.8	<0.001
60–64	64.1	0.6	70.2	0.8	6.0	<0.0001
65–69	64.4	0.6	65.6	0.7	1.2	0.05
70–74	61.6	0.7	63.2	0.7	1.6	0.04
75–79	59.2	0.9	59.5	0.7	0.4	0.63
80–84	54.5	1.5	57.2	1.0	2.8	0.49
85+	54.8	2.7	56.7	1.6	1.9	0.35
**Overall** *	**61.9**	**0.3**	**64.5**	**0.3**	**2.6**	**<0.0001**

RS: relative survival point estimate, SE: standard error of the estimate.

* Age-adjusted relative survival, testing performed using age-adjusted model.

Table 3 shows age-specific sex differences in relative survival, considering separately risk of death in first year after diagnosis and in the following 4 years. Relative survival decreased with age during the first year after diagnosis (89.0% to 75.9% in men, 92.1% to 74.6% in women). By contrast, the 5-year relative survival conditional on surviving one year was very similar across age groups. Except for the oldest age group, relative survival was higher in women both during the first year (significant in patients aged 15–74) and during 2^nd^ to 5^th^ year (significant in age groups 15–44 and 55–64).

**Table 3:** Age-specific 1-year relative survival and 5-year relative survival conditional on surviving one year, period 2002–2006, by sex.

		Men		Women		Women –Men	
	Age	RS, %	SE	RS, %	SE	Difference	*P-*value
1-year	15–44	89.0	0.8	92.1	0.8	3.1	<0.01
relativesurvival	45–54	88.6	0.5	91.0	0.5	2.4	<0.01
	55–64	88.3	0.3	90.8	0.3	2.5	<0.0001
	65–74	85.3	0.3	86.7	0.3	1.4	<0.001
	75+	75.9	0.4	74.6	0.3	−1.3	0.02
2nd to 5thyear	15–44	73.7	1.5	78.0	1.4	4.3	0.01
conditionalsurvival	45–54	72.1	0.9	73.9	1.0	1.8	0.29
	55–64	72.9	0.5	76.7	0.6	3.8	<0.0001
	65–74	73.9	0.5	74.3	0.5	0.4	0.74
	75+	75.1	0.9	77.2	0.7	2.1	0.35

RS: relative survival point estimate, SE: standard error of the estimate.

We further used model-based period analysis to summarize sex differences in relative survival providing relative excess risk (RER) of dying from CRC (women vs. men). A substantial survival advantage of women was observed among subjects under 65 years of age (RER 0.84, 95% CI 0.80–0.87), but not in older subjects (RER 0.98, 95% CI 0.95–1.01). To evaluate the contribution of potential stage and subsite distribution differences between sexes, we added these variables in the multivariate model. Age-specific results were practically unchanged by the adjustment for both younger (RER 0.86, 95% CI 0.82–0.90) and older subjects (RER 1.01, 95% CI 0.98–1.04). Findings were similar when we excluded the first year of follow-up from the analyses and considered only excess mortality in the 2^nd^ to 5^th^ year. Resulting RERs were 0.91 (95% CI 0.86–0.96) in younger and 1.02 (95% CI 0.97–1.07) in older subjects.

Table 4 shows sex differences in 5-year relative survival in subgroups by age and stage (model results in Table S1). The prognosis was substantially better in patients with early disease and was worsening with increasing age in both sexes. Relative survival of patients with localized disease was consistently higher in women, regardless of age. When looking at patients with regional or advanced disease, women had a survival advantage only under 65 years of age (borderline significant or nonsignificant in patients aged 45–54 years). With sex differences of 3.3, 2.0 and 8.4 percent units and RERs of 0.59, 0.72, and 0.76, the survival advantage of women was most pronounced in patients below 45 years of age with localized, regional or advanced disease, respectively. By contrast, worse survival was noted for women among patients with advanced disease aged over 75 years (RER 1.13, *P*-value<0.0001). Results of patients with unreported CRC stage show similar sex differences as those for staged patients.

**Table 4:** Sex differences in 5-year relative survival of colorectal patients for subgroups by stage and age.

		Men		Women		Women-Men	Multivariate model	
Stage	Age	RS, %	SE	RS, %	SE	Difference	RER, 95% CI	*P-*value
Localized	15–44	90.7	1.9	94.0	1.6	3.3	0.59 (0.49–0.72)	<0.0001
	45–54	90.3	1.2	92.4	1.2	2.1	0.73 (0.63–0.85)	<0.0001
	55–64	90.9	0.7	92.6	0.8	1.7	0.72 (0.64–0.82)	<0.0001
	65–74	88.3	0.8	88.5	0.8	0.1	0.81 (0.72–0.92)	<0.01
	75+	83.3	1.6	90.2	1.2	6.9	0.88 (0.78–1.00)	0.05
Regional	15–44	71.7	3.3	73.7	3.3	2.0	0.72 (0.61–0.85)	<0.0001
	45–54	68.1	1.9	71.0	2.3	2.9	0.90 (0.80–1.00)	0.05
	55–64	66.6	1.2	72.3	1.4	5.7	0.88 (0.81–0.96)	<0.01
	65–74	66.7	1.2	63.8	1.2	−2.8	0.99 (0.92–1.07)	0.88
	75+	61.0	2.0	58.2	1.5	−2.8	1.08 (1.00–1.16)	0.06
Advanced	15–44	19.6	2.8	28.0	3.2	8.4	0.76 (0.65–0.88)	<0.001
	45–54	18.3	1.5	20.4	1.9	2.1	0.94 (0.86–1.03)	0.18
	55–64	16.5	0.9	18.4	1.3	1.9	0.93 (0.87–0.98)	0.01
	65–74	14.8	0.8	13.1	0.9	−1.8	1.04 (0.99–1.10)	0.11
	75+	10.1	1.1	9.5	0.8	−0.5	1.13 (1.07–1.18)	<0.0001
Not reported	15–44	72.4	2.5	83.6	2.1	11.2	0.66 (0.56–0.77)	<0.0001
	45–54	71.3	1.5	76.4	1.6	5.1	0.82 (0.74–0.90)	<0.0001
	55–64	68.3	0.8	73.4	1.0	5.1	0.81 (0.76–0.86)	<0.0001
	65–74	62.9	0.8	68.1	0.8	5.2	0.91 (0.86–0.95)	<0.001
	75+	56.0	1.1	55.5	0.8	−0.5	0.98 (0.93–1.03)	0.42

RS: relative survival point estimate, SE: standard error of the estimate, RER: relative excess risk women vs. men, CI: confidence interval.

* adjusting for age, stage and subsite, including interaction of sex with age and sex with stage.

Estimates of relative excess risk (RER) associated with female sex were derived using multivariate model*.

Sex differences in 5-year relative survival in subgroups by age and subsite are shown in Table 5 (model results in Table S2). Again, most of relative survival estimates were higher in women, with the exception of left colon and rectum cancer patients aged over 75 years. The multivariate model showed similar patterns of age-specific survival advantages for female patients across CRC subsites, suggesting independence of disease localization of survival advantages of women (*P*-value for interaction term 0.12).

**Table 5:** Sex differences in 5-year relative survival of colorectal patients for subgroups by subsite and age.

		Men		Women		Women-Men	Multivariate model	
Subsite	Age	RS, %	SE	RS, %	SE	Difference	RER, 95% CI	*P-*value
Right colon	15–44	64.9	3.1	68.1	3.1	3.1	0.70 (0.59–0.82)	<0.0001
	45–54	61.7	2.0	64.1	2.2	2.4	0.86 (0.78–0.95)	<0.01
	55–64	62.1	1.2	65.7	1.3	3.6	0.84 (0.79–0.91)	<0.0001
	65–74	63.7	1.0	64.3	0.9	0.6	0.94 (0.88–0.99)	0.03
	75+	61.5	1.4	64.3	0.9	2.8	1.01 (0.95–1.06)	0.82
Left colon	15–44	63.5	3.1	70.2	2.7	6.7	0.75 (0.63–0.88)	<0.001
	45–54	65.4	1.7	67.9	1.8	2.6	0.92 (0.83–1.02)	0.11
	55–64	69.7	0.9	71.9	1.1	2.1	0.90 (0.84–0.97)	<0.01
	65–74	65.9	0.8	67.1	0.9	1.2	1.00 (0.94–1.06)	0.97
	75+	58.9	1.4	58.6	1.1	−0.3	1.08 (1.02–1.14)	0.01
Rectum	15–44	65.4	2.3	72.5	2.6	7.1	0.73 (0.62–0.86)	<0.001
	45–54	64.8	1.2	68.8	1.6	3.9	0.90 (0.82–0.99)	0.03
	55–64	63.0	0.8	71.7	0.9	8.7	0.88 (0.83–0.94)	<0.0001
	65–74	62.0	0.8	63.8	0.9	1.8	0.98 (0.93–1.03)	0.40
	75+	53.1	1.3	51.4	1.0	−1.7	1.05 (0.99–1.11)	0.08

RS: relative survival point estimate, SE: standard error of the estimate, RER: relative excess risk women vs. men, CI: confidence interval.

* adjusting for age, stage and subsite, including interaction of sex with age and sex with subsite.

Estimates of relative excess risk (RER) associated with female sex were derived using multivariate model* (‘Colon - unspecified/other’ subsite cases were omitted).

## Discussion

To our knowledge, this is to date the largest study specifically focusing on sex differences in CRC survival and the first one utilizing multivariate population-based relative survival analysis including CRC stage and disease subsite. We found an overall survival advantage of female CRC patients compared to male patients. The difference in 5-year relative survival was most pronounced in patients under 65 years of age, with the exception of patients aged 45–49 years. Excess risk of death was 14% lower in young (under 65 years of age) women compared to men even after adjustments for stage and subsite. Lower excess risk of CRC death was not limited to the first year after diagnosis, but persisted also during subsequent years. Among patients with localized disease, a survival advantage of women was seen at all ages. By contrast, the survival advantage of women was confined to patients below 65 years of age when the diagnosis was made at a regional or advanced stage. Among patients with advanced stage CRC, older women (≥75 years) had significantly worse prognosis than men. Sex differences in survival did not vary by location of CRC.

Previous evidence on importance of sex as a CRC prognostic factor has been inconclusive. Two hospital-based studies reported higher disease-specific survival following colorectal surgery in women [4], [5]. The EUROCARE-4 study, which reported 5-year relative survival of cancer patients in European countries diagnosed between 1995 and 1999, found higher survival in women in most sites, including colon and rectum. However, no comparison was provided adjusting for strong prognostic factors, such as CRC stage and subsite [31]. Studies examining patients diagnosed in the 1980s or earlier, including a EUROCARE high resolution study on 5-year relative survival [32], mostly had not shown any differences in survival between sexes [6], and it was suggested that the differences were actually arising and widening in recent years [7]. Recent population-based studies observed higher cancer-specific survival in younger (i.e. individuals aged below 45 [7] or 50 [8] years) women compared to men, but equal or worse survival in older women compared to men. In our study, the survival advantage of female patients was still substantial among individuals aged 60–64.

We have found substantial differences between subgroups by CRC stage in older patients. Whereas survival in patients with localised cancers was higher in women, survival from metastatic CRC was higher in men. Worse cancer-specific survival in women compared to men was previously noted among older patients in an Australian study; however, no interaction between sex and clinical stage was found [8]. Other studies reported better survival for females and also addressed a possible modifying effect of stage. The results are, however, conflicting. One of these studies did not find variation of sex differences by stage [3]. Other studies reported higher survival in female patients for CRC stage I or IV only [5], or for stage II only [12].

Female sex hormones decrease the risk of CRC, and postmenopausal women have increased risk of colon cancer compared to premenopausal women of the same age [33]. This effect is not limited to endogenous hormones, as use of oral contraceptives [34] and postmenopausal hormonal replacement therapy (HRT) seem to also protect women from CRC. Women using HRT were shown to be at lower risk of CRC in a meta-analysis of 18 epidemiologic studies [35] and in a randomized trial [36]. Most notably, HRT use was also shown to extend survival in CRC patients [37], [38], [39].

A protective effect of endogenous female sex hormones may explain the superior survival of young women compared to men. Surprisingly, we did not find higher survival in female patients 45–49 compared to males. According to a national German survey performed in 2003, this was the age group when most of women started HRT [40]. A possible explanation for the survival patterns observed in our study might be that lower oestrogen exposition in perimenopausal period before inception of HRT might have accounted for the weak survival advantage of women aged 45–49; more frequent use in older women might have contributed to the stronger survival benefit of older postmenopausal women. Three other German surveys performed between 1997 and 2003 [41] showed that current HRT use sharply dropped after 65 years of age, which corresponds well with sex differences patterns observed in our study.

Higher survival of women with early CRC has previously been noted in a study examining a cohort of patients after colorectal surgery. It has been hypothesized that influences of sex hormones on the immune system may be the reason for observed differences, as testosterone has a detrimental effect, and female hormones have stimulatory effects on immunological response [5]. The observed patterns are consistent with this immunological hypothesis, as patients with early CRC don’t regularly undergo adjuvant treatment and their immunological barrier is thought to be usually sufficient to cope with cancer spread [5]. Paulson and colleagues indeed showed that the survival advantage of women is limited to patients without adjuvant therapy [3]. Lower survival may also be associated with an ongoing inflammatory response, with elevated C-reactive protein, which is seen more often in males [42] and which reflect an increased number of deaths from cardiovascular diseases mainly in men [4], [43]. Oestrogen furthermore seems to play role in protection of female cancer patients against cardiac mass loss [44].

To answer the question, whether higher survival of women could be partially explained by use of screening offers by individuals over 50 (likely reflected in CRC stage distribution), and differences in subsite distribution of CRC, we fitted a further multivariate model and adjusted for potentially different proportions of early or left-sided tumours. However, estimates of relative excess risk barely changed when we included these variables in our multivariate model, suggesting an at best very modest contribution to the higher survival in young women. Early post-operative mortality also seems to explain only a small part of the advantage. Our modelling results rather point to long-term decreased excess risk of death in younger and middle-aged women, possibly due to sustained protective effects of female sex hormones.

Most studies assessing the difference between men and women in CRC survival estimated either overall or cancer-specific survival. Overall survival considers deaths from all causes and is therefore affected by comorbidities, which may considerably differ between men and women. This estimate is therefore usually accompanied by cancer-specific survival; however, inaccurate classification of the cause of death may still confound the results [6]. In our study, we assessed relative survival, which is the preferred and most widely used measure of cancer outcomes in population-based registries. It provides information on the excess mortality associated with CRC irrespective whether it is directly or indirectly related to the disease. Relative survival has the advantage to be independent of recorded cause of death, and it also takes deaths from cardiovascular events possibly associated with CRC diagnosis or treatment into account.

A major limitation of our study is the lack of specific indicators of hormonal status, such as information on menopausal status, HRT or use of oral contraceptives which prohibited a more direct assessment of their impact. However, this limitation is shared with most registry based studies. Similarly, we were not able to include information on a given CRC treatment, which is usually not available in population-based registries [45]. There was a substantial proportion of cases without complete information on CRC stage. Nevertheless, we were still able to include almost 100,000 patients with recorded CRC stage from different German federal states in the multivariate analysis. Furthermore, lack of stage information is mostly due to registry practice, and unlikely to be a source of selection bias resulting from differential registration practices for men and women. This assumption is supported by the similarity of age specific sex differences in staged and unstaged patients.

In conclusion, our large population-based study confirmed a CRC survival advantage of women compared to men in Germany. Higher female 5-year age-adjusted relative survival compared to men was most pronounced in young and middle aged patients and patients with localized disease. Differences in survival between sexes were not restricted to early postoperative mortality, and they were not explained by different distribution of CRC stages or subsites. Taken together, our results support the hypothesis that sex hormones might be a plausible explanation of better CRC prognosis among young female patients. In our study, women aged 50–64 also had higher relative survival than male patients of the same age, which might be explained by notable exposure of German women to HRT during the study period. Efforts should be made to further elucidate the reasons underlying the survival advantages of women among CRC patients and their potential implications for clinical practice.

## Supporting Information

Table S1 Estimates of regression coefficients and respective relative excess risk (RER) in multivariate model adjusting for age, stage and subsite, including interaction of sex with age and sex with stage.
(DOC)

Table S2 Estimates of regression coefficients and respective relative excess risk (RER) in multivariate model adjusting for adjusting for age, stage and subsite, including interaction of sex with age and sex with subsite.
(DOC)
