# Supplementary material for: Sex Differences in Colorectal Cancer Survival: Population-Based Analysis of 164,996 Colorectal Cancer Patients in Germany
Source: PLoS One. 2013 Jul 5;8(7):e68077. doi: 10.1371/journal.pone.0068077 (PMC3702575; doi:10.1371/journal.pone.0068077)
Supplement: Table S1 — Estimates of regression coefficients and respective relative excess risk (RER) in multivariate model adjusting for age, stage and subsite, including interaction of sex with age and sex with stage. (DOC) [file pone.0068077.s001.doc]

|  | **Coefficient estimate** | **RER** | **95% CI** | ***P*-value** |
| --- | --- | --- | --- | --- |
| **Age** |  |  |  |  |
| 15-44 |  | 1.00 |  |  |
| 45-54 | 0.03 | 1.04 | 0.92-1.16 | 0.55 |
| 55-64 | 0.09 | 1.10 | 0.99-1.22 | 0.08 |
| 65-74 | 0.25 | 1.28 | 1.16-1.42 | <0.0001 |
| 75+ | 0.63 | 1.88 | 1.70-2.09 | <0.0001 |
| **Sex** |  |  |  |  |
| Male |  | 1.00 |  |  |
| Female | -0.53 | 0.59 | 0.49-0.72 | <0.0001 |
| **Interaction age-sex(female)** | | |  |  |
| 15-44 |  | 1.00 |  |  |
| 45-54 | 0.22 | 1.24 | 1.04-1.48 | 0.02 |
| 55-64 | 0.20 | 1.22 | 1.04-1.44 | 0.01 |
| 65-74 | 0.32 | 1.38 | 1.18-1.61 | <0.0001 |
| 75+ | 0.40 | 1.49 | 1.27-1.74 | <0.0001 |
| **Stage** |  |  |  |  |
| Localized |  | 1.00 |  |  |
| Regional | 1.21 | 3.35 | 3.05-3.68 | <0.0001 |
| Advanced | 2.77 | 15.93 | 14.64-17.32 | <0.0001 |
| Not reported | 1.48 | 4.39 | 4.04-4.78 | <0.0001 |
| **Interaction sex(female)-Stage** | | |  |  |
| Localized |  | 1.00 |  |  |
| Regional | 0.20 | 1.22 | 1.06-1.40 | <0.01 |
| Advanced | 0.25 | 1.28 | 1.13-1.45 | <0.001 |
| Not reported | 0.11 | 1.11 | 0.98-1.27 | 0.10 |
| **Subsite** |  |  |  |  |
| Right colon |  | 1.00 |  |  |
| Left colon | -0.13 | 0.88 | 0.85-0.91 | <0.0001 |
| Colon-unspecified/other | 0.13 | 1.14 | 1.09-1.19 | <0.0001 |
| Rectum | 0.01 | 1.01 | 0.98-1.04 | 0.44 |
| **Follow-up year** |  |  |  |  |
| 1 | 0.89 | 2.43 | 2.27-2.60 | <0.0001 |
| 2 | 0.58 | 1.78 | 1.66-1.91 | <0.0001 |
| 3 | 0.44 | 1.55 | 1.44-1.67 | <0.0001 |
| 4 | 0.22 | 1.25 | 1.15-1.35 | <0.0001 |
| 5 |  | 1.00 |  |  |

RER: relative excess risk, CI: confidence interval
