# Supplementary material for: Sex Differences in Colorectal Cancer Survival: Population-Based Analysis of 164,996 Colorectal Cancer Patients in Germany
Source: PLoS One. 2013 Jul 5;8(7):e68077. doi: 10.1371/journal.pone.0068077 (PMC3702575; doi:10.1371/journal.pone.0068077)
Supplement: Table S2 — Estimates of regression coefficients and respective relative excess risk (RER) in multivariate model adjusting for adjusting for age, stage and subsite, including interaction of sex with age and sex with subsite. (DOC) [file pone.0068077.s002.doc]

|  | **Coefficient estimate** | **RER** | **95% CI** | ***P*-value** |
| --- | --- | --- | --- | --- |
| **Age** |  |  |  |  |
| 15-44 |  | 1.00 |  |  |
| 45-54 | -0.02 | 0.99 | 0.87-1.11 | 0.81 |
| 55-64 | 0.05 | 1.05 | 0.94-1.17 | 0.41 |
| 65-74 | 0.21 | 1.23 | 1.11-1.37 | <0.001 |
| 75+ | 0.59 | 1.81 | 1.62-2.02 | <0.0001 |
| **Sex** |  |  |  |  |
| Male |  | 1.00 |  |  |
| Female | -0.36 | 0.70 | 0.59-0.82 | <0.0001 |
| **Interaction age-sex(female)** | | |  |  |
| 15-44 |  | 1.00 |  |  |
| 45-54 | 0.21 | 1.23 | 1.03-1.48 | 0.03 |
| 55-64 | 0.19 | 1.21 | 1.02-1.43 | 0.03 |
| 65-74 | 0.29 | 1.34 | 1.14-1.58 | <0.001 |
| 75+ | 0.37 | 1.44 | 1.22-1.70 | <0.0001 |
| **Subsite** |  |  |  |  |
| Right colon |  | 1.00 |  |  |
| Left colon | -0.16 | 0.85 | 0.81-0.89 | <0.0001 |
| Rectum | -0.01 | 0.99 | 0.95-1.03 | 0.60 |
| **Interaction sex(female)-subsite** | | |  |  |
| Right colon |  | 1.00 |  |  |
| Left colon | 0.07 | 1.07 | 1.00-1.14 | 0.04 |
| Rectum | 0.04 | 1.04 | 0.98-1.11 | 0.16 |
| **Stage** |  |  |  |  |
| Localized |  | 1.00 |  |  |
| Regional | 1.31 | 3.70 | 3.44-3.97 | <0.0001 |
| Advanced | 2.90 | 18.13 | 16.99-19.35 | <0.0001 |
| Not reported | 1.52 | 4.56 | 4.27-4.87 | <0.0001 |
| **Follow-up year** | |  |  |  |
| 1 | 0.83 | 2.30 | 2.14-2.47 | <0.0001 |
| 2 | 0.55 | 1.73 | 1.61-1.86 | <0.0001 |
| 3 | 0.43 | 1.53 | 1.42-1.65 | <0.0001 |
| 4 | 0.22 | 1.24 | 1.14-1.35 | <0.0001 |
| 5 |  | 1.00 |  |  |

RER: relative excess risk, CI: confidence interval
